# Therapeutic Mammoplasty in Breast Cancer Surgery: Expanding the Boundaries of Breast Conservation Through Oncoplastic Integration

**DOI:** 10.7759/cureus.107373

**Published:** 2026-04-20

**Authors:** Sofia I Balamoti, Kyriaki Alevrogianni, Konstantinos Benetatos

**Affiliations:** 1 Breast Surgery, Athens Bioclinic Hospital, Athens, GRC; 2 Plastic and Reconstructive Surgery, 401 General Military Hospital of Athens, Athens, GRC

**Keywords:** aesthetic outcomes, breast cancer, breast-conserving surgery, oncoplastic breast surgery, therapeutic mammoplasty

## Abstract

Breast-conserving surgery is a standard treatment for early-stage breast cancer, providing oncologic outcomes comparable to mastectomy. However, its use is limited in patients with larger tumors or unfavorable tumor-to-breast ratios, where adequate resection may compromise aesthetic results. Therapeutic mammoplasty has emerged as an oncoplastic approach that combines tumor excision with reduction-based reconstruction, allowing wider resections while preserving breast shape.

This study was conducted as a structured narrative review to provide a clinically relevant synthesis of the available evidence on therapeutic mammoplasty. Given the heterogeneity of the literature, a qualitative approach was adopted, focusing on oncologic safety, surgical feasibility, indications, and aesthetic and patient-reported outcomes.

The available evidence indicates that therapeutic mammoplasty achieves oncologic outcomes comparable to standard breast-conserving surgery, with low rates of positive margins and acceptable local recurrence. The ability to perform wider resections expands the indications for breast conservation, particularly in patients who might otherwise require mastectomy. Improved aesthetic outcomes and high levels of patient satisfaction are consistently reported, and, in selected patients, additional functional benefits may be observed.

Therapeutic mammoplasty represents an effective oncoplastic strategy that extends the applicability of breast-conserving surgery while maintaining oncologic safety and improving patient-centered outcomes. Its role is expected to expand as integrated oncologic and reconstructive approaches become increasingly adopted in modern breast cancer care.

## Introduction and background

Breast cancer surgery has undergone substantial evolution over the past century, moving away from highly radical ablative procedures toward more conservative and patient-centered approaches that aim to preserve oncologic safety while also maintaining postoperative quality of life and taking into consideration the cosmetic result [1]. The early dominance of radical mastectomy, as described by Halsted, was driven by the concept of achieving maximal local disease control, which frequently came at the cost of considerable physical and psychological morbidity. As understanding of tumor biology has deepened and advances in imaging, systemic therapies, and multidisciplinary care have been progressively incorporated into practice, this approach has gradually shifted. Breast-conserving surgery combined with radiotherapy is now widely established as a standard treatment option for early-stage disease [2]. Long-term randomized evidence has consistently demonstrated equivalent overall survival between breast-conserving surgery and mastectomy, and this has broadened the definition of surgical success to include not only disease control but also outcomes such as cosmesis, body image, and psychosocial well-being.

Despite these advances, conventional breast-conserving surgery remains limited, as achieving an optimal balance between adequate oncologic margins and preservation of breast aesthetics is often challenging. From a clinical perspective, the volume of tissue excised relative to total breast volume represents a key determinant of postoperative deformity. Resections exceeding approximately 20% are frequently associated with visible contour irregularities, asymmetry, and reduced patient satisfaction [3]. This limitation becomes more evident in patients with unfavorable tumor-to-breast ratios, multifocal disease, or tumors located in cosmetically sensitive areas such as the lower pole, medial quadrants, or the retroareolar region, where even relatively small excisions may lead to a disproportionate aesthetic compromise. In routine clinical practice, this often results in patients being directed toward mastectomy, not because of oncologic necessity, but due to the anticipated cosmetic outcome.

The development of oncoplastic breast surgery represents an important step in addressing these limitations, and it effectively bridges oncologic resection with reconstructive principles. By incorporating plastic surgical techniques into oncologic procedures, oncoplastic approaches allow for wider excisions while maintaining, and in many cases improving, aesthetic outcomes [4]. These techniques can be broadly divided into volume displacement and volume replacement strategies. Volume displacement relies on local tissue rearrangement and glandular reshaping, and it often incorporates reduction mammoplasty or mastopexy patterns, whereas volume replacement involves the use of autologous tissue transferred from distant donor sites to restore breast volume [5]. The choice between these approaches depends on a combination of tumor characteristics, breast morphology, patient expectations, and surgeon experience, which reflects the increasingly tailor-made approach of modern breast cancer care.

Within this framework, therapeutic mammoplasty has emerged as one of the most versatile and clinically impactful oncoplastic techniques. By combining wide local excision with established reduction mammoplasty principles, it enables the resection of larger tumor volumes while simultaneously reshaping the breast and, when appropriate, achieving contralateral symmetrization [6]. From an operative perspective, this approach not only fulfills oncologic requirements but also anticipates and addresses the aesthetic consequences of surgery. In this way, a potential deformity is effectively transformed into an opportunity for improved breast contour and symmetry. As a result, the indications for breast conservation have been expanded, particularly in patients with larger tumors, ptotic breasts, or complex tumor locations, and it allows many of these patients to avoid mastectomy without compromising oncologic safety [7].

Beyond its technical benefits, therapeutic mammoplasty reflects a broader shift in surgical philosophy from a purely ablative model to one that is reconstructive and restorative. In this context, success is no longer defined solely by margin status or recurrence rates, but also by the extent to which surgery preserves or enhances the patient’s body image and overall quality of life. Emerging evidence supports this perspective and demonstrates high levels of patient satisfaction, improved psychosocial outcomes, and favorable quality-of-life measures following oncoplastic procedures compared with more traditional techniques. In addition, therapeutic mammoplasty may reduce the need for secondary corrective procedures and facilitate long-term symmetry, and both are important factors in patient satisfaction and healthcare resource utilization.

Nevertheless, despite its growing adoption and encouraging outcomes, therapeutic mammoplasty remains variably implemented across institutions and lacks a standardized framework to guide its indications, technique selection, and integration into treatment pathways [8]. Much of the current literature focuses on technical descriptions and retrospective analyses, while there is relatively limited emphasis on its strategic role within the broader context of breast cancer surgery. As a result, it is still frequently regarded as an adjunctive option rather than as a central component of modern breast-conserving therapy.

In light of these gaps, a more structured and conceptually driven synthesis of the available evidence on therapeutic mammoplasty is needed. Such a perspective should move beyond the evaluation of oncologic safety and aesthetic outcomes, and clarify the role of therapeutic mammoplasty within the broader surgical management of breast cancer. In particular, it is important to consider how it contributes to expanding the indications for breast conservation in routine clinical practice. Accordingly, the aim of the present study is to provide a comprehensive review of therapeutic mammoplasty, examining its surgical principles, indications, oncologic outcomes, and aesthetic benefits while proposing a framework that supports its integration as a core component of contemporary breast cancer management, rather than viewing it solely as a reconstructive adjunct.

## Review

Methods

The present study was conducted as a structured narrative review with the aim of providing a clinically relevant synthesis of the available evidence on therapeutic mammoplasty within the broader context of oncoplastic breast-conserving surgery. A narrative approach was selected instead of a formal systematic review or meta-analysis due to the heterogeneity of the existing literature, which includes randomized controlled trials, prospective cohort studies, retrospective series, and technique-oriented publications. This variability in study design and reporting has been consistently highlighted in previous reviews of oncoplastic breast surgery, where differences in outcome definitions and surgical techniques limit the feasibility of quantitative pooling and support the use of qualitative synthesis methods [9].

The literature search was conducted using major electronic databases (e.g., PubMed and Scopus), focusing on studies relevant to therapeutic mammoplasty and oncoplastic breast surgery published in English. These references encompass landmark trials that established the oncologic safety of breast-conserving therapy as well as key publications describing the evolution of oncoplastic techniques. Particular attention was given to studies examining the integration of reconstructive principles into oncologic surgery, as these have played a central role in expanding the indications for breast conservation beyond traditional constraints [10].

Eligibility for inclusion was based on clinical relevance to the aims of the study rather than strict methodological criteria, consistent with the narrative design. Studies were included if they addressed at least one of three main domains, namely oncologic outcomes, surgical technique and feasibility, and aesthetic or patient-reported outcomes. Oncologic outcomes included parameters such as margin status, local recurrence, and survival. Surgical feasibility referred to descriptions of therapeutic mammoplasty and related oncoplastic procedures, particularly those involving reduction mammoplasty and volume displacement strategies. Aesthetic and patient-reported outcomes encompassed both objective assessments of breast symmetry and subjective measures of patient satisfaction and quality of life, reflecting the increasing importance of these endpoints in contemporary breast cancer care [11].

Studies that focused exclusively on mastectomy without a reconstructive comparator, purely technical descriptions without associated clinical outcomes, or non-clinical experimental work were excluded, as they did not contribute directly to the evaluation of therapeutic mammoplasty within a clinical decision-making context. Greater emphasis was placed on studies comparing therapeutic mammoplasty with standard breast-conserving surgery or mastectomy with reconstruction, as such comparisons are essential in defining its role as an alternative surgical approach [12].

Data extraction was performed through a detailed qualitative assessment of each included study, focusing on study design, patient characteristics, tumor features, surgical technique, and reported outcomes. Given the variability in reporting across studies, including differences in complication definitions, methods of cosmetic evaluation, and duration of follow-up, a quantitative meta-analysis was not considered appropriate. Instead, a thematic synthesis was conducted, consistent with previous work evaluating oncoplastic outcomes across heterogeneous datasets [13]. The extracted data were subsequently organized into three main analytical domains, namely oncologic safety, surgical feasibility and indications, and aesthetic and patient-reported outcomes, reflecting key priorities for patients with breast cancer in the current decade. This structure reflects the core elements of surgical decision-making in breast cancer treatment, where oncologic adequacy must be balanced with technical feasibility and expected cosmetic outcomes. Such a framework has been widely applied in the evaluation of oncoplastic surgery and facilitates comparison between studies while enhancing clinical applicability [14].

**Figure 1:**
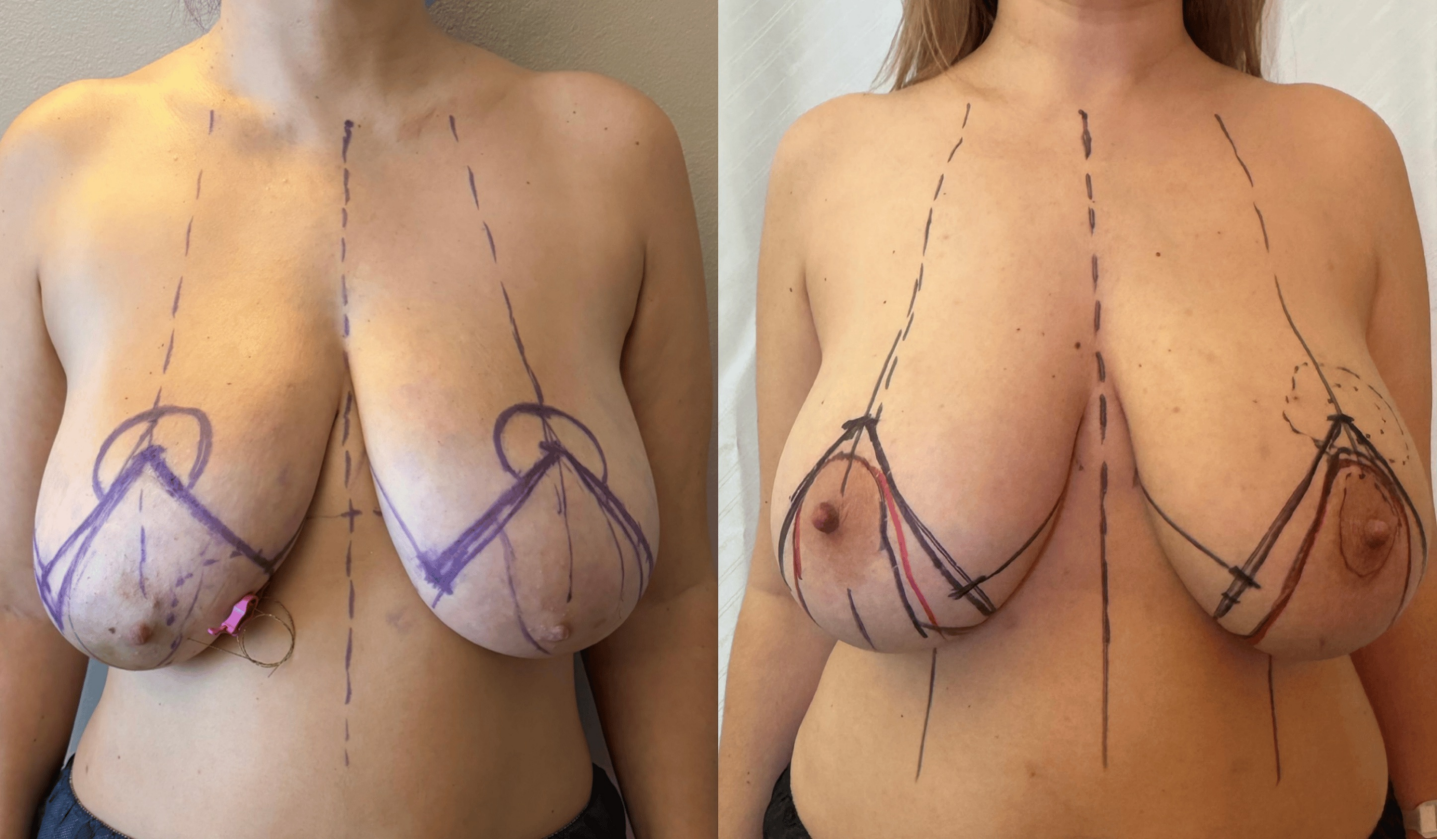
Preoperative surgical markings in two different patients undergoing therapeutic mammoplasty. Preoperative planning in two separate patients demonstrates variation in incision pattern and pedicle selection according to tumor location, breast morphology, and anticipated resection volume, highlighting the adaptability of reduction-based oncoplastic techniques. Images are author-generated and used with patient consent. No identifiable patient information is disclosed.

Alongside the literature synthesis, schematic illustrations were used to clarify how therapeutic mammoplasty is planned and performed in practice. These diagrams focus on the key steps of the procedure, particularly how oncologic resection is combined with reduction mammoplasty techniques. As shown in Figure 1, this includes pedicle selection and tissue rearrangement, and highlights how the approach can be adapted depending on tumor location and breast anatomy [15].

This study is primarily a narrative review and includes illustrative clinical images. Written informed consent was obtained from all patients for the use and publication of their images. No identifiable patient information is disclosed. According to institutional policy, formal ethical approval was not required for this type of study.

Results

Taken together, the available studies suggest that therapeutic mammoplasty achieves oncologic outcomes comparable to standard breast-conserving surgery, while allowing breast preservation in cases where this would not previously have been considered feasible. The oncologic safety of breast-conserving surgery itself is already well established, with landmark randomized trials showing no difference in overall survival between breast-conserving surgery with radiotherapy and mastectomy [16]. In this setting, therapeutic mammoplasty can be seen less as a separate approach and more as a practical way of extending these established principles to more complex clinical situations.

In everyday practice, one of the main advantages of reduction-based oncoplastic techniques is the ability to remove larger volumes of tissue without compromising margin status. This becomes particularly relevant when tumor size, location, or multifocality would otherwise limit the use of standard breast-conserving surgery. Several series report low rates of positive margins and fewer re-excisions, which likely reflects the fact that once reshaping is part of the plan, the surgeon is less constrained by concerns about the final breast contour [17]. At the same time, reported local recurrence rates remain within the expected range for conventional techniques, which supports the oncologic reliability of this approach [18].

Margin control remains central to surgical decision-making, and therapeutic mammoplasty appears to facilitate wider excisions while maintaining clear margins. This is particularly useful in patients with larger tumors or less favorable tumor locations, where standard techniques may lead to unacceptable cosmetic outcomes. Available data suggest that wider resections achieved in this context improve surgical confidence without adding morbidity [19]. The adoption of standardized margin definitions has also contributed to reinforcing the safety of breast-conserving strategies, even when larger resections are performed [20].

Surgical Feasibility and Expansion of Indications

A recurring finding across the literature is that therapeutic mammoplasty expands the group of patients who can be offered breast conservation. Patients who might previously have been directed toward mastectomy because of tumor size relative to breast volume, multifocal disease, or tumor position can now often be managed with an oncoplastic approach. By redistributing the remaining breast tissue after excision, reduction-based techniques help preserve breast shape and symmetry [21]. In practical terms, the decision between breast conservation and mastectomy is no longer driven only by tumor characteristics, but also by whether an acceptable reconstruction can be achieved at the same time [22].

Another point worth noting is the adaptability of the technique. Pedicle choice can be adjusted according to tumor location, allowing preservation of vascular supply while maintaining aesthetic outcome. Studies looking at feasibility report high rates of successful tumor excision together with acceptable complication rates, suggesting that the technique can be integrated into routine practice without major limitations [23]. At the same time, performing contralateral symmetrization during the same procedure can improve overall balance, particularly in patients with pre-existing asymmetry or larger breast volume [24].

Aesthetic Outcomes and Patient-Reported Outcomes

The difference compared with standard breast-conserving surgery becomes most apparent when looking at aesthetic outcomes. Larger resections performed without reconstruction often result in visible deformity, whereas therapeutic mammoplasty incorporates reshaping as part of the procedure. This allows preservation and, in some cases, improvement of breast appearance [25]. Patient-reported outcomes consistently show high satisfaction, with improvements in body image and overall quality of life [26]. Objective assessments using standardized tools support these findings and demonstrate consistent results across different techniques and patient groups [27].

**Figure 2:**
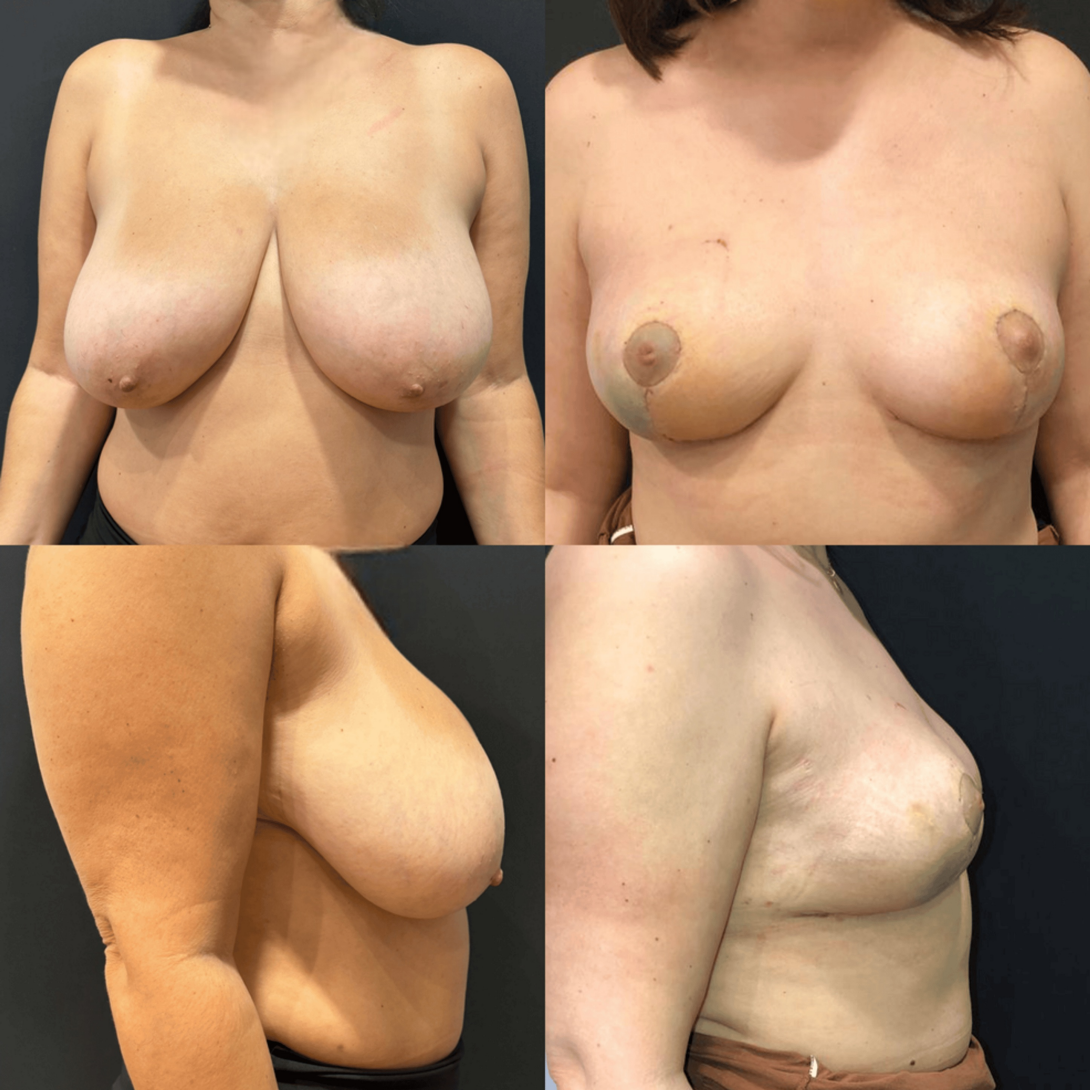
Preoperative and postoperative clinical photographs demonstrating outcomes following therapeutic mammoplasty. Preoperative frontal and lateral views demonstrate macromastia and ptosis. Postoperative images show restoration of breast contour, elevation of the nipple-areola complex, and improved symmetry following wide local excision and reduction-based reconstruction. Images are author-generated and used with patient consent. No identifiable patient information is disclosed.

**Figure 3:**
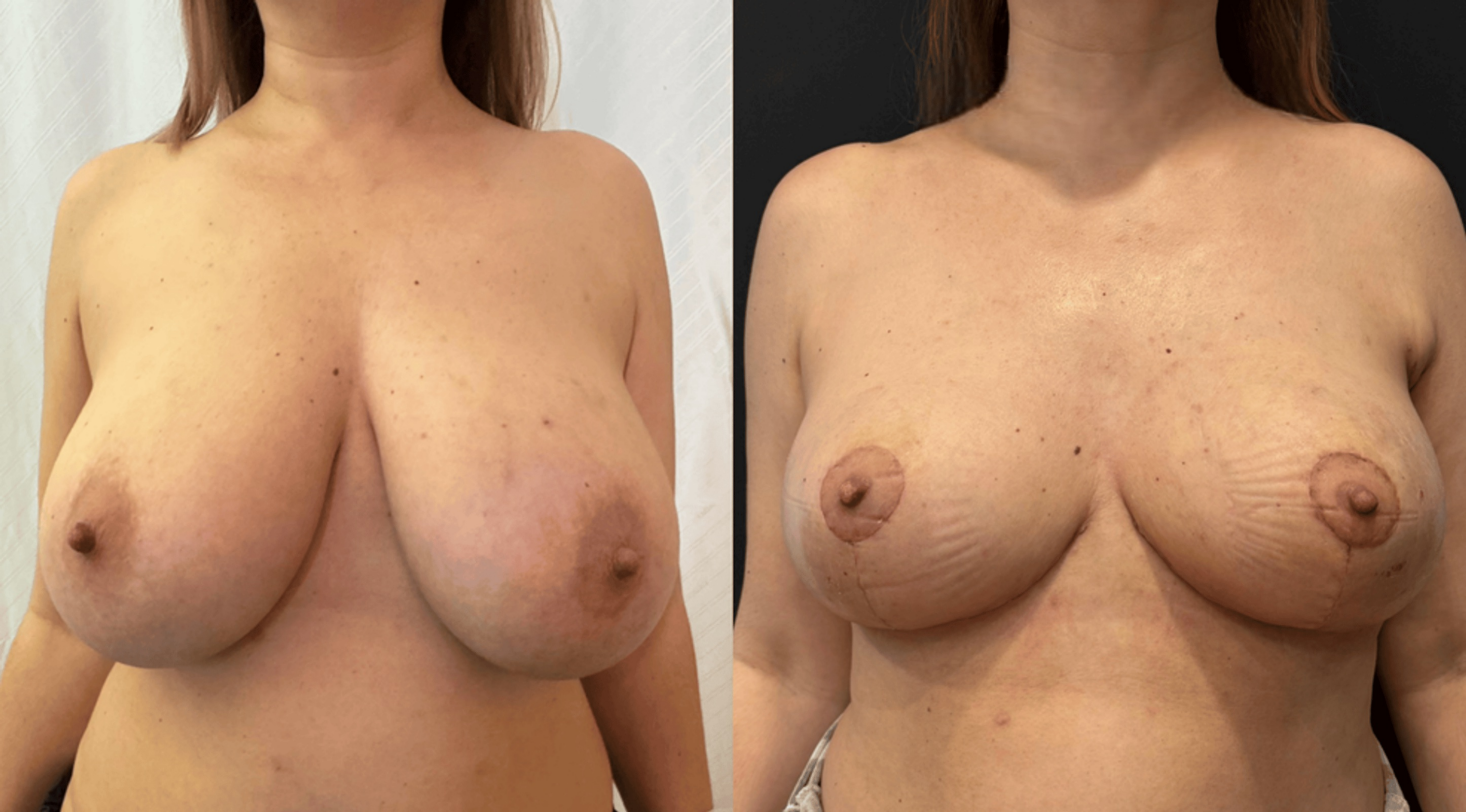
Preoperative and postoperative clinical photographs demonstrating outcomes in a second patient undergoing therapeutic mammoplasty. Preoperative view shows ptotic breasts with excess volume. Postoperative appearance demonstrates successful breast conservation with improved contour, repositioning of the nipple-areola complex, and satisfactory symmetry following tumor excision and immediate reconstruction. Images are author-generated and used with patient consent. No identifiable patient information is disclosed.

Representative clinical cases demonstrating preoperative and postoperative outcomes following therapeutic mammoplasty are presented in Figure 2 and Figure 3, illustrating the ability of the technique to maintain breast contour, symmetry, and aesthetic integrity following wide local excision.

In addition to aesthetic effects, there may also be a functional benefit in selected patients. In women with larger breasts, reduction can relieve symptoms such as back and neck discomfort. This combination of oncologic treatment and functional improvement adds to the overall value of the procedure and supports its role in contemporary breast surgery [28].

Complications and Impact on Adjuvant Therapy

In terms of complications, therapeutic mammoplasty appears largely comparable to standard breast-conserving surgery. Most reported events are minor in nature and include delayed wound healing, fat necrosis, and localized infection, which are generally manageable with conservative measures and rarely require reoperation. Rates of major complications remain low in most series, supporting the overall safety profile of the technique. Importantly, these events do not appear to adversely affect long-term oncologic outcomes. Furthermore, the available evidence suggests that the use of oncoplastic techniques does not result in clinically meaningful delays in the initiation of adjuvant treatments, including radiotherapy or systemic therapy, which remains a critical consideration in breast cancer management [29].

When compared with mastectomy followed by reconstruction, therapeutic mammoplasty often results in a simpler overall treatment pathway. Reconstruction, particularly when involving autologous flaps, may require multiple stages and is associated with a higher risk of complications, including flap-related morbidity or failure. In contrast, therapeutic mammoplasty is usually completed in a single procedure, reducing the need for additional interventions and potentially lessening the overall burden on both patients and healthcare systems [30].

Comparative Effectiveness

Comparative data further support the role of therapeutic mammoplasty. When compared with mastectomy and immediate reconstruction, it offers similar oncologic outcomes while achieving higher patient satisfaction and more favorable aesthetic results [31]. Preservation of native breast tissue combined with immediate reshaping contributes to a more natural result and may reduce psychological impact.

Addressing oncologic and aesthetic considerations at the same time also reduces the need for delayed corrective procedures, which are frequently required after standard breast-conserving surgery. In this sense, therapeutic mammoplasty represents a more integrated and patient-focused approach, aligning surgical planning with long-term quality-of-life outcomes [32].

Discussion

The present review highlights therapeutic mammoplasty as a pivotal evolution in breast cancer surgery, representing not merely a refinement of technique but a fundamental shift in surgical strategy. Although the oncologic safety of breast-conserving surgery is no longer in question, the way surgery is planned has changed considerably with the incorporation of reconstructive principles. In practice, this means that instead of accepting the cosmetic limitations imposed by tumor excision, the resection itself can be planned within a reconstructive framework. Therapeutic mammoplasty reflects this shift, allowing oncologic resection to be performed with adequate margins while, at the same time, preserving or even improving breast shape [33]. More broadly, this change mirrors the move in surgical oncology toward approaches that place greater emphasis on the overall patient outcome. One of the clearest consequences of this shift is the expansion of breast conservation. In the past, factors such as tumor size relative to breast volume, multifocal disease, or unfavorable location often led to mastectomy, even when there was no strict oncologic indication. With the introduction of reduction-based oncoplastic techniques, these limitations have become less restrictive. Wider resections can be performed, and the remaining tissue can be reshaped immediately, which allows patients who would previously have required a mastectomy to be treated conservatively [34]. In clinical terms, this represents an important step toward more individualized surgical decision-making, in which treatment is tailored to each patient’s oncologic profile, anatomical characteristics, and personal preferences. The longstanding tension between achieving clear margins and maintaining breast shape is also addressed in this setting. With conventional breast-conserving surgery, this balance can be difficult, particularly in anatomically challenging areas. Therapeutic mammoplasty changes this dynamic by allowing more extensive excision without an obvious aesthetic penalty. As a result, the surgeon is less constrained by concerns about postoperative appearance and can focus on achieving complete tumor removal [35]. Current margin guidelines support this approach and reinforce the oncologic safety of performing larger resections when needed [36].

Beyond oncologic considerations, the effect on patient-reported outcomes is significant. The aesthetic result after surgery is closely linked to body image, psychological recovery, and overall quality of life. Evidence from oncoplastic series consistently shows higher levels of patient satisfaction compared with standard breast-conserving surgery or mastectomy [37]. In addition, reduction-based techniques may offer functional benefits in patients with larger breasts, including relief of physical symptoms such as back and neck discomfort, which further contributes to overall well-being [38]. When considered alongside mastectomy with reconstruction, therapeutic mammoplasty offers a different treatment pathway. Although reconstructive techniques have improved, they are often associated with multiple procedures and a higher complication burden. In contrast, therapeutic mammoplasty is usually performed in a single stage, which simplifies treatment and reduces the need for additional interventions [39]. The preservation of native breast tissue also contributes to outcomes that tend to feel more natural, both in appearance and function.

There are, however, practical challenges that need to be taken into account. One of the most commonly discussed issues is the effect of tissue rearrangement on radiotherapy planning. Displacement of the tumor bed can make localization more difficult. In practice, this problem can be addressed with intraoperative clip placement and modern imaging techniques, which allow accurate targeting of the treatment area even after extensive rearrangement [40]. This highlights the importance of coordination within the multidisciplinary team. Another point relates to the variability of surgical technique. Therapeutic mammoplasty includes a wide range of approaches with different pedicle choices and incision patterns, reflecting its adaptability. While this flexibility is one of its strengths, it also makes comparison between studies more difficult and introduces variability in reported outcomes [41]. For this reason, efforts toward standardization and structured training are likely to be important for the future development of the field. Appropriate patient selection remains essential, as optimal outcomes depend on careful consideration of tumor characteristics, breast size and morphology, patient comorbidities, and individual expectations regarding aesthetic results. Several studies emphasize the importance of individualized planning and a multidisciplinary approach when selecting the most suitable surgical strategy [42]. Further work in this area may help refine selection criteria and support more consistent decision-making.

From a wider perspective, therapeutic mammoplasty represents a convergence of oncologic and reconstructive thinking. Effective collaboration between breast surgeons, plastic surgeons, radiologists, and radiation oncologists is central to achieving good outcomes, particularly in more complex cases [43]. This model is in line with current trends in personalized care, where treatment is adapted not only to the disease but also to patient-specific factors. Ongoing developments are also likely to influence the future role of therapeutic mammoplasty. Advances in imaging, surgical planning, and intraoperative techniques are expected to improve both precision and reproducibility. At the same time, increasing emphasis on patient-reported outcomes and value-based care will continue to favor approaches that address both oncologic and aesthetic goals [44]. There is also evidence that the impact of therapeutic mammoplasty extends beyond individual patients. By making breast conservation feasible in a wider range of cases, it may contribute to reducing overall mastectomy rates and aligning surgical practice more closely with patient preferences [45]. This has potential implications at a system level, particularly in relation to resource use associated with multi-stage reconstruction.

The successful adoption of these techniques also depends on appropriate training. Therapeutic mammoplasty requires familiarity with both oncologic and reconstructive principles, which underlines the need for dedicated training pathways and ongoing skill development [46]. The introduction of structured curricula and competency-based assessment may help support wider and more consistent implementation. In parallel, the use of standardized outcome measures will be important. Incorporating validated aesthetic assessment tools and patient-reported outcome measures can improve the quality of evidence and allow for more meaningful comparisons between studies [47]. This will be essential in establishing clearer guidelines for clinical practice. Future research should aim to address current limitations, including the need for prospective data, longer follow-up, and direct comparisons between techniques. The integration of new technologies such as three-dimensional imaging and advanced planning tools may further improve surgical accuracy and predictability [48]. In summary, therapeutic mammoplasty can be considered a key development in breast cancer surgery. By enabling wider excision while preserving breast shape and expanding the indications for breast conservation, it offers a practical solution to the balance between oncologic safety and aesthetic outcome, and continues to reshape the role of breast-conserving surgery in modern practice [49].

To improve clarity and allow direct comparison between studies, the key characteristics of the included literature are summarized in Table 1 and Table 2, which present study design, patient population, surgical technique, and principal oncologic and aesthetic outcomes.​​​​​​​​​​​​

**Table 1:** Clinical studies. BCS: Breast-Conserving Surgery; OPS: Oncoplastic Surgery; IBR: Immediate Breast Reconstruction

Author (Year)	Study Type	Sample Size	Technique Classification	Population	Oncologic Outcomes	Aesthetic/Clinical Outcomes
Veronesi et al. (2002) [1]	Randomized controlled trial	701	Conventional BCS vs mastectomy	Early-stage breast cancer	Equivalent overall survival	Not primary endpoint
Fisher et al. (2002) [2]	Randomized controlled trial	1851	Lumpectomy ± radiotherapy vs mastectomy	Invasive breast cancer	Equivalent overall survival	Not primary endpoint
Currie et al. (2013) [7]	Cohort study	20	Level II OPS	Large/ptotic breasts	Low recurrence rates	Acceptable cosmesis
Potter et al. (2020) [12]	Prospective multicenter cohort	2916	Level II OPS (therapeutic mammoplasty) vs mastectomy ± IBR	Breast cancer	No delay in adjuvant therapy, low re-excision rates	Lower complication rates vs mastectomy
O’Connell et al. (2018) [16]	Prospective cohort	880	Level II OPS (therapeutic mammoplasty)	Breast cancer	Low re-excision rates, high margin negativity	Acceptable complication rates
Bamford et al. (2015) [17]	Cohort study	68	Level II OPS	Breast cancer	Low local recurrence rates	Good cosmetic outcomes
McCulley and Macmillan (2005) [21]	Case series	50	Reduction-based therapeutic mammoplasty	Breast cancer	Wide excision with clear margins	Symmetry achieved
Kelemen et al. (2019) [22]	Retrospective cohort	190	Level II OPS (wise-pattern mammoplasty)	Breast cancer	High rate of negative margins	Good symmetry outcomes
Refaat et al. (2020) [23]	Cohort study	144	Level II OPS (round block technique)	Early-stage breast cancer	High margin negativity	88.8% good-excellent cosmetic outcomes
McCulley et al. (2006) [24]	Case series	11	Central therapeutic mammoplasty	Central breast tumors	Negative margins achieved	Acceptable cosmetic outcomes
Gulis et al. (2021) [25]	Retrospective cohort	146	Bilateral therapeutic mammoplasty	Breast cancer	Low recurrence rates	High symmetry scores
Stein et al. (2020) [26]	Retrospective cohort	249	Level II OPS vs mastectomy with reconstruction	Breast cancer	Comparable oncologic outcomes	Improved quality of life, fewer revisions
Majdak-Paredes et al. (2015) [29]	Cohort study	98	Therapeutic mammoplasty with intraoperative imaging	Breast cancer	Reduced re-excision rates	Improved surgical precision
Tenofsky et al. (2014) [30]	Cohort study	142	Oncoplastic BCS vs standard BCS	Breast cancer	Equivalent oncologic safety	Comparable complication rates
Burrah et al. (2020) [31]	Cohort study	270	Level II OPS (round block mammoplasty)	Breast cancer	Low recurrence rates	Good cosmetic outcomes
Blok et al. (2022) [37]	Prospective cohort	75	Oncoplastic BCS	Breast cancer	Oncologically safe resection	High patient satisfaction
Regaño et al. (2009) [43]	Cohort study	23	Oncoplastic BCS after neoadjuvant therapy	Locally advanced breast cancer	Negative margins achieved	Good cosmetic outcomes

**Table 2:** Reviews, guidelines, and conceptual papers. OPS: Oncoplastic Surgery; TM: Therapeutic Mammoplasty; DCIS: Ductal Carcinoma In Situ

Author (Year)	Article Type	Focus	Key Contribution
Rainsbury (2007) [3]	Narrative review	Oncoplastic principles	Defined reconstructive approach to breast-conserving surgery
Clough et al. (2003) [4]	Clinical study	Oncoplastic techniques	Introduced the modern oncoplastic surgery concept
Clough et al. (2010) [5]	Review/classification	OPS classification	Established Level I and II framework
Macmillan et al. (2014) [6]	Narrative review	Therapeutic mammoplasty	Technical overview and indications
McIntosh and O'Donoghue (2012) [8]	Systematic review	Therapeutic mammoplasty outcomes	Evidence synthesis of clinical results
Haloua et al. (2013) [9]	Systematic review	Oncoplastic surgery	Identified limitations and research gaps
Chatterjee et al. (2019) [10]	Consensus statement	OPS definition	Standardized terminology and classification
De La Cruz et al. (2016) [11]	Systematic review	OPS outcomes	Large pooled outcome analysis
Nanda et al. (2021) [13]	Cochrane systematic review	Oncoplastic surgery	High-level evidence assessment
Gilmour et al. (2021) [14]	Clinical guideline	Best practice in OPS	Recommendations for clinical implementation
Kronowitz et al. (2007) [15]	Technical guideline	Reconstruction techniques	Practical guidance for defect repair
Moran et al. (2014) [20]	Consensus guideline	Surgical margins	Defined “no ink on tumor” standard
Cardoso et al. (2007) [27]	Methodological study	Cosmetic assessment	Introduced BCCT.core evaluation tool
Munhoz et al. (2013) [28]	Narrative review	OPS techniques	Detailed indications and approaches
Clough et al. (2018) [32]	Long-term cohort study	OPS outcomes	Demonstrated long-term oncologic safety
Patel et al. (2019) [33]	Narrative review	OPS techniques	Practical surgical framework
Grubnik et al. (2013) [34]	Review	Therapeutic mammoplasty	Clinical and aesthetic outcomes
Losken et al. (2014) [35]	Review	Margin control	Highlighted oncologic advantages of OPS
Morrow et al. (2016) [36]	Consensus guideline	Margin standards (DCIS)	Standardized margin definitions
Iwuchukwu et al. (2012) [38]	Narrative review	Role of TM	Expansion of breast conservation
Yoon et al. (2016) [40]	Systematic review	OPS and radiotherapy	Integrated outcome analysis
Kijima et al. (2016) [41]	Case series (technical)	TM technique	Nipple-areola grafting application

## Conclusions

Therapeutic mammoplasty represents an important step forward in the surgical management of breast cancer, mainly because it brings oncologic resection and reconstruction together in a single procedure. The findings of this study suggest that it achieves oncologic outcomes comparable to standard breast-conserving surgery, while, at the same time, offering better aesthetic results and high patient satisfaction. In practice, what changes is how the balance between tumor clearance and breast shape is handled. Surgeons are no longer forced to compromise. Wider excisions can be performed, and the breast can still be reshaped. A practical consequence is that more patients can now be considered for breast conservation. In clinical practice, this is particularly evident in patients with larger tumors or less favorable tumor-to-breast ratios, who, in the past, would often be directed toward mastectomy. With oncoplastic techniques, this is not always necessary. Therapeutic mammoplasty, therefore, acts not only as a technique but also as a way of changing how surgical decisions are made. The effect on patients should not be underestimated. Preserving the breast while maintaining or improving its shape has a direct impact on body image and confidence. This is something that becomes very clear in follow-up. In selected cases, especially in patients with larger breasts, the reduction component may also relieve symptoms such as back or neck discomfort. This adds a functional benefit on top of the oncologic one.

From a practical point of view, the procedure can also simplify treatment. It is usually performed in a single stage, which reduces the need for additional operations. This is relevant both for patients and for healthcare systems, where reducing the number of procedures matters. At the same time, there are limitations. Patient selection remains important, and the technique requires appropriate experience. Not all centers apply it in the same way, and there is still variability in technique. This highlights the need for better standardization and structured training. In addition, more consistent reporting of outcomes, including patient-reported measures, would help strengthen the evidence. Looking ahead, further improvements in imaging and surgical planning are likely to make the procedure more precise and easier to reproduce. As breast cancer care continues to evolve, approaches that combine oncologic safety with acceptable aesthetic outcomes will become increasingly important. Therapeutic mammoplasty fits well within this direction. In summary, therapeutic mammoplasty should be seen as more than a reconstructive option. It changes the way breast-conserving surgery can be applied, expands the number of patients who can benefit from it, and maintains oncologic safety while improving aesthetic and functional outcomes. Its role in everyday practice is likely to continue growing as experience increases and techniques become more standardized.
